# Optimum Thermal Processing for Extended Shelf-Life (ESL) Milk

**DOI:** 10.3390/foods6110102

**Published:** 2017-11-20

**Authors:** Hilton Deeth

**Affiliations:** School of Agriculture and Food Sciences, University of Queensland, Brisbane 4072, Australia; h.deeth@uq.edu.au; Tel.: +61-7-38708251

**Keywords:** extended shelf-life, ESL milk, psychrotrophic spore-formers, B*-value, β-lactoglobulin denaturation

## Abstract

Extended shelf-life (ESL) or ultra-pasteurized milk is produced by thermal processing using conditions between those used for traditional high-temperature, short-time (HTST) pasteurization and those used for ultra-high-temperature (UHT) sterilization. It should have a refrigerated shelf-life of more than 30 days. To achieve this, the thermal processing has to be quite intense. The challenge is to produce a product that has high bacteriological quality and safety but also very good organoleptic characteristics. Hence the two major aims in producing ESL milk are to inactivate all vegetative bacteria and spores of psychrotrophic bacteria, and to cause minimal chemical change that can result in cooked flavor development. The first aim is focused on inactivation of spores of psychrotrophic bacteria, especially *Bacillus cereus* because some strains of this organism are pathogenic, some can grow at ≤7 °C and cause spoilage of milk, and the spores of some strains are very heat-resistant. The second aim is minimizing denaturation of β-lactoglobulin (β-Lg) as the extent of denaturation is strongly correlated with the production of volatile sulfur compounds that cause cooked flavor. It is proposed that the heating should have a bactericidal effect, B* (inactivation of thermophilic spores), of >0.3 and cause ≤50% denaturation of β-Lg. This can be best achieved by heating at high temperature for a short holding time using direct heating, and aseptically packaging the product.

## 1. Introduction

Extended shelf-life (ESL) milk has gained substantial market share in many countries [1]. It has a refrigerated shelf-life of 21–45 days with some manufacturers claiming a shelf-life of up to 90 days. It is produced by two principal technologies: (1) Thermal processing using more severe conditions than pasteurization but less severe than ultra-high-temperature (UHT) processing; and (2) Non-thermal processes such as microfiltration [2] and bactofugation [3], usually combined with a final thermal pasteurization treatment to meet regulatory requirements. Only the thermal processing option is discussed here. 

Thermally produced ESL milk has been reviewed previously [1]. This review is based on information recently published in a book by Deeth and Lewis [4]. 

## 2. Heating Methods

The heating systems used for ESL processing are of two major types, direct and indirect. In direct systems, heating occurs through direct contact between steam and the product and in indirect systems the heat is transferred to the product from steam or hot water through a stainless steel barrier in a heat exchanger. These systems are described in numerous publications, e.g., [4,5,6,7], and will be discussed here only briefly in relation to their effects on the quality of ESL milk.

### 2.1. Direct Heating

In direct heating processes, the milk is first heated indirectly in a plate or tubular heat exchanger to 70–80 °C and then heated to the required high temperature by direct contact with dry culinary steam. The milk is held at the required temperature for the required period of time while it passes through a holding tube. The heated milk then passes to a vacuum chamber, which removes the water from the condensed steam and cools the milk to approximately the same temperature to which it was pre-heated prior to the steam heating stage. The milk is then cooled indirectly to ~4 °C. 

There are two modes of steam heating, steam injection and steam infusion. In milk processing, these are often described as steam-into-milk and milk-into-steam. They differ considerably in terms of equipment but ESL milk produced by the two methods is very similar, although some authors have reported advantages of steam infusion over steam injection [1]. 

The major distinguishing feature of direct heating methods relevant to ESL milk processing is the high rate of heating and cooling, on the order of 0.5 s for a temperature change of 50–60 °C. 

### 2.2. Indirect Heating

Indirect heating involves the use of plate or tubular heat exchangers for all heating and cooling stages. Of note in this regard is that a considerable amount of heat (up to ~90%) can be recovered by using the heat in the hot milk, after the holding tube, to heat the incoming cold milk. The heat recovery is greater than for direct systems, where it is ~50%. This is obviously an economic advantage of indirect systems. 

The rates of heating and cooling in the high-temperature sections of indirect systems are much slower than in direct systems. This has an important consequence for ESL processing because, for the same bactericidal effect, the indirect systems cause more chemical change than do direct systems. Thus more cooked flavor is produced in indirect systems than in direct systems for the same bacterial, including spore, inactivation.

### 2.3. Nominal Temperature–Time Combinations

For direct systems, the only heat input of relevance to spore inactivation occurs between the pre-heat temperature (70–80 °C) and the highest temperature reached, both in the heating and cooling stages, and, because heating and cooling are so rapid, this heat input is very close to what occurs in the holding tube only. However, for indirect systems the heat input of interest includes that to which the milk is subjected in the heating and cooling stages as well as the holding tube. 

Heating conditions are usually described in terms of a temperature–time combination, e.g., 125 °C for 5 s. This condition applies to the holding tube only and hence, for ESL milk, closely reflects the heat input in a direct heating system but considerably underestimates the heat input of interest in an indirect heating system. This was well illustrated by Rysstad and Kolstad [1] who showed that for a direct and an indirect process having equal bactericidal effects (the nominal temperature–time combinations were 135 °C for 0.5 s and 127 °C for 1 s, respectively), the direct system caused much less chemical change than the indirect system. The lower temperature in the indirect system is compensated for by the extra heat input from the upper heating and cooling sections to achieve the desired bactericidal effect.

The bactericidal effect is usually expressed in terms of either B* or *F*_0_. B* refers to inactivation of thermophilic spores; a process with B* = 1 causes a 9-log reduction of the spores and is equivalent to holding the product at 135 °C for 10.1 s. *F*_0_ refers to inactivation of *Clostridium botulinum* spores; a process with *F*_0_ = 2.8 causes a 12-log reduction of the spores and is equivalent to holding the product at 121.1 °C for 2.8 min.

The key aim for optimizing the heating conditions for ESL processing is to maximize the sporicidal effect but minimize the chemical effect, which results in cooked flavor production. For this, direct heating systems are more appropriate than indirect systems.

### 2.4. Alternative Direct Heating Systems

#### 2.4.1. The Pure-Lac™ System

This system (SPX FLOW, Charlotte, NC, USA), based on steam infusion, was developed to produce ESL and UHT milk with a taste similar to that of pasteurized milk but to effect a high level of spore inactivation. The system also includes specially developed packaging equipment that ensures post-processing contamination (PPC) is minimal. The temperature–time conditions for the holding tube are given as 130–145 °C for <1 s [1,8]. This is a very broad temperature range and the effects of heating at 130 °C and 145 °C are very different, with the lower temperature being marginal for inactivating spores. In the publication by Fredsted et al. [8], it is unclear what temperature was used to produce the ESL milk on which the bacteriological, chemical, and sensory analyses were performed. A 2008 publication [9] states that the temperature for the Pure-Lac process can be “as high as 140 °C”; however, a temperature–time profile given for the Pure-Lac process indicates a temperature of 135 °C [9] (p. 23). 

The short holding time is a central feature of the system. Precise control of this is achieved by imposing sufficient backpressure on the holding tube to ensure single-phase turbulent flow. 

#### 2.4.2. The Innovative Steam Injection (ISI) Process

Huijs et al. [10] reported the development of Innovative Steam Injection (ISI) heating, which can heat milk at 150–180 °C for <0.1 s. The process achieved a very high bacterial kill and was capable of producing commercially sterile milk with minimal chemical change; it caused only 20–25% denaturation of β-Lg. However, it did not inactivate the native milk protease, plasmin, and in UHT milk stored at 20 °C, bitterness due to plasmin-induced proteolysis resulted in a short shelf-life. However, for milk stored at 7 °C, no proteolysis occurred during storage for 28 days because plasmin has very low activity at this low temperature [11]. Therefore ISI technology is applicable to ESL milk without an additional pre-heating step to inactivate plasmin, which is necessary for UHT milk [12].

### 2.5. The Millisecond Technologies (MST) Process

A patented technology involving both temperature and pressure manipulation has been proposed for extending the shelf-life of pasteurized milk [13,14]. According to Millisecond Technologies Corporation, the commercial suppliers of the equipment, the process for extending the shelf-life of milk operates at <70 °C for <1 s and achieves a bacterial kill of ≥8 logs and a shelf-life of 45–60+ days. This was compared with HTST pasteurization at 72–80 °C for 15 s, which produces milk with a shelf-life of 14–18 days (www.millisecondtechnologies.com). However, Myer et al. [14] reported somewhat different information. In their most successful trial, milk, pasteurized at 73.8 °C, entered an MST chamber at 73 °C and exited the chamber at 75.2 or 78.5 °C. Bacterial counts for these milks were reported to be <10 CFU/mL after storage for 63 days at 4 °C. However, *Bacillus* and *Paenibacillus* species were isolated after this treatment indicating that spores were not inactivated by the treatment. 

According to the patent [13], the destruction of bacteria by the MST process is partly by heat and partly by the rapid “pressure variation” of at least 10^5^ Pa/s. Milk is sprayed under pressure (800,000 Pa according to Myer et al. [14]) in droplets of ≤0.3 mm into a heated MST chamber under vacuum (~0.025 Pa). The milk is rapidly heated in the chamber to the desired temperature (in <0.02 s). The bacterial kill is largely attributable to sudden decompression in the MST chamber. Rapid decompression has previously been reported to inactivate vegetative bacteria [15] and even heat-tolerant spores [16]. 

MST technology has potential for processing milk although, as Myer et al. [14] commented, several parameters of the process need to be examined including the efficacy of the process to inactivate more thermally robust microorganisms such as *Bacillus* species. Until the technology can be shown to inactivate spores of psychrotrophic spore-forming bacteria, it has limited application for production of ESL milk with a long shelf-life.

## 3. Microbiological Considerations 

There are two major aspects to be considered when optimizing the conditions for producing ESL milk, microbiological and sensory. Ideally, ESL milk should not show any microbial growth during refrigerated storage. However, this is generally not the case because of several factors.

The microbiological issues can be divided into those related to the bactericidal nature of the heating conditions and those associated with post-processing contamination. The relative importance of these for the shelf-life of ESL milk depends on whether the milk is packaged aseptically or under very clean, but not aseptic, conditions. The nature of the heating conditions and storage temperature are the only factors relevant to the microbiology of aseptically packaged milk, while post-processing contamination together with the heating and storage conditions is important for ESL milks not packaged aseptically. Since ESL milk is usually not packaged under aseptic conditions, the bacteria in such ESL milk include spore-forming bacteria whose spores are not destroyed by the heating process, as well as spore-forming and non-spore-forming bacteria entering the milk after processing. 

A further consideration that can affect ESL milk is the bacteriological quality of the raw milk. Because of the logarithmic reduction of bacterial counts by heat, the higher the bacterial count in the raw milk, the higher will be the residual count in the heated milk. Also, if psychrotrophic bacteria such as Pseudomonads are allowed to grow in the raw milk and produce heat-resistant proteases, bitter flavors can develop in the ESL milk during storage. As a rule of thumb, the total count in the raw milk should not exceed 10^5^ CFU/mL.

### 3.1. Microbiological Issues Related to the Heating Process

Bacterial growth in ESL milk is by psychrotrophic organisms only as ESL milk is stored under refrigeration. Since ESL heating at ≥120 °C destroys all non-spore-forming bacteria but does not destroy all spores, psychrotrophic spore-formers are a major concern. They are the only bacteria that cause spoilage when post-processing contamination is eliminated, as in aseptically packaged milk, provided the storage temperature is kept at less than ~6 °C. The ESL heat treatment can also activate spores to germinate and thus allow their vegetative cells to grow during storage. 

There have been a small number of investigations on the spore population of ESL milks collected or packaged under aseptic conditions. Mayr et al. [17] aseptically collected milk samples from a commercial direct processing plant operated at 127 °C for 5 s. They found that *B. licheniformis* (the dominant organism at 73% of isolates), followed by *B. subtilis*, *B. cereus*, *Brevibacillus brevis*, and *B. pumilus*, were the most common spore-formers. When the ESL milks were held at 10 °C for 23 weeks, no growth occurred, indicating the absence of psychrotrophic spore-formers. However, when the milks were incubated at 30 °C, growth occurred. Bacteria isolated from the 30 °C incubated milks were found to be able to grow at 8 or 10 °C, suggesting some spores were sub-lethally injured during the heat treatment and were revived at 30 °C. *B. cereus* was the most psychrotrophic of the spore-formers isolated. Blake et al. [18] found *B. licheniformis*, *B. insolitus*, *B. coagulans*, and *B. cereus/thuringiensis* in poor-quality milk (total count of >10^8^ CFU/mL) directly heated at 120–132 °C for 4 s and packaged aseptically. No organisms grew in milk processed at temperatures ≥134 °C for 4 s. In a trial with good-quality milk, Blake et al. [18] observed no psychrotrophic growth in milks processed at ≥128 °C. Although not dealing with ESL milk as it is known today, the study of Cromie et al. [19] is instructive. They found that *B. circulans* was the dominant spoilage organism in milks heated at 72 to 88 °C for 15 s, aseptically packaged and stored at 3 or 7 °C for up to seven weeks. A similar observation was reported by Rysstad and Kolstad [1], where the total bacterial count of aseptically packaged pasteurized milk stored at 6 °C reached 10^6^ CFU/mL (a nominal spoilage level) after 40 days. They also reported that when ESL milk produced by the Pure-Lac™ system (see Section 2.4.1) was packaged aseptically, a shelf-life at the “relatively abusive temperature” of 10 °C of ≥45 days was achieved [1].

#### 3.1.1. Psychrotrophic Spore-Formers

In determining the ideal temperature–time combinations of ESL milk processing to ensure a long shelf-life, a major consideration is choosing combinations that destroy spores of psychrotrophic bacteria. Spores of psychrotrophic bacteria are generally more heat-sensitive than those of mesophilic and thermophilic bacteria and hence less severe conditions will be required to destroy the former, although there are exceptions, e.g., some *Paenibacillus* species and some strains of *B. cereus*. In this regard, processing of ESL milk differs from UHT processing, in which the aim is to destroy the spores of mesophilic and thermophilic bacteria. In fact, the accepted minimum requirement for UHT processing to produce “commercially sterile” milk is a 9-log reduction of the thermophilic spore count; this is equivalent to heating at 135 °C for 10.1 s. For ESL milk, the suggested corresponding criterion is a 6-log reduction of the psychrotrophic spore count [8]. However, the conditions used for producing ESL milk seldom meet this criterion. 

The numbers and types of spores differ considerably between raw milk samples. This is largely due to the different environmental conditions of the cows as most spores enter the milk from teat and udder surfaces during milking. High spore numbers are found in very dry dusty conditions, wet and muddy conditions, and in the environs of housed animals, particularly when cows are fed silage. Thus the spore counts in raw milk from unhoused, pasture-grazing cows are typically <10^2^ CFU/mL but 10^3^ CFU/mL in milk from housed cows. Furthermore, the proportion of the spores that will germinate and grow under refrigeration conditions—that is, the proportion that is psychrotrophic—also varies. Reported percentages of raw milk samples containing psychrotrophic spore-formers vary from 25% to 83% [20]. 

Variation in the types of spores may be due to differences in environmental conditions. For example, Blake et al. [18] carried out research on ESL milk in Logan, UT, USA, where the ambient temperatures are low, and suggested that their results on the heating conditions for inactivating psychrotrophic spores may have been influenced by acclimatization of the spores to cold environmental conditions. This may suggest that if conditions for producing ESL milk are set to those found by Blake et al. [18] to produce ESL milk with a long shelf-life, they should be suitable for all ESL milk production and may in fact incorporate a margin of safety (see Section 5).

*Paenibacillus* and *B. cereus* are spore-formers of particular relevance to ESL milk. These are discussed in the following sections.

#### 3.1.2. *Paenibacillus*

*Paenibacillus*, which was formerly part of the genus *Bacillus*, has emerged in recent years as an organism of concern to the dairy industry. It has been found in silage and feed concentrates and can enter the milk from these sources. Doll et al. [21] reported it to be the major organism in raw bulk tank milk (48% of isolates). It has also been reported to be a major spoilage bacterium in pasteurized milk in New York State [22,23] and in ESL milk in South Africa [24]. It can cause bitterness due to its production of proteases [25]. Scheldeman et al. [26] reported *Paenibacillus* spores in UHT milk, suggesting its resistance to UHT processing. 

*Paenibacillus* species have a broad range of growth temperatures from ~5 to 55 °C. Their growth in pasteurized and ESL milk is evidence of their growth at low temperature, but Scheldeman et al. [26] reported they could grow at temperatures as high as 55 °C. The optimum growth temperatures of *Paenibacillus* species range from 28 to 42 °C [27]. Therefore, *Paenibacillus* can be psychrotrophic, mesophilic, or thermophilic and produce heat-resistant spores. This is an unusual combination of properties and an unfortunate one for the dairy industry as it indicates some strains of these organisms can survive all common milk heat treatments and grow at both refrigeration and ambient temperatures. 

#### 3.1.3. *B. cereus*

Apart from the fact that the growth of psychrotrophic spore-formers can limit the shelf-life of ESL milk, some psychrotrophic spore-formers are pathogenic and hence pose a safety issue in ESL milk. The major organism of concern is *Bacillus cereus*, although some authors have also included other *Bacillus* species such as *B. circulans* as potential pathogens. *B. cereus* is a major spoilage organism in pasteurized [11] and ESL [21,28] milk. Some strains are pathogenic and the spores of some are quite heat-resistant. For these reasons, this organism is particularly relevant to ESL milk and is discussed in some detail in the following section. 

Various aspects of *B. cereus* that are relevant to ESL milk are discussed in this section. There is a vast amount of information on this organism in the literature and so this section is in no way comprehensive. The importance of *B. cereus* in the dairy/food industry is indicated by the reviews that have appeared on it, e.g., [29,30,31,32,33]. The reader is referred to these for further information. 

● Incidence: 

*B. cereus* is widespread in the environment and is a common contaminant of milk and milk products [34,35]. It has some closely related species; in fact, the *B. cereus* group (sometimes referred to as *B.* *cereus sensu lato*) comprises eight species: *B. cereus* (*B. cereus sensu strict*), *B. anthracis*, *B. thuringiensis*, *B. mycoides*, *B. pseudomycoides*, *B. weihenstephanensis*, *B. toyonensis*, and *B. cytotoxicus* [33], which are difficult to distinguish phenotypically [36]. It can therefore be assumed that in most cases where “*B. cereus*” has been isolated from milk and milk-based products, it could include other members of the *B. cereus* group. Using 16S rRNA gene sequence data of dairy isolates, Ivy et al. [37] identified *B*. *cereus*, *B. weihenstephanensis*, and *B. mycoides* from the *B. cereus* group. It is interesting to note that *B. thuringiensis* has been isolated from UHT milk. This organism is used as a biological control agent and could be in high concentrations in some agricultural environments and animal feed sources. Furthermore, *B. toyonensis* is used as a feed additive [33]. 

Several surveys of the incidence of *B. cereus* in raw and pasteurized milk and milk-based products have been conducted, with the percentage of *B. cereus-*positive samples ranging from very low to 100% [38,39,40,41]. Its incidence in raw and pasteurized milk is commonly 20–60%. Griffiths and Phillips [42] reported that three studies found it to be the main psychrotrophic spore-former in raw milk and others have found it to be the dominant psychrotrophic spore-former in pasteurized milk, e.g., [38,43,44]. In general, the counts of *B. cereus* in raw milk are low, <100 CFU/mL [45], often <1 CFU/mL [46]. 

Like other spore-formers, *B. cereus* can enter milk from numerous sources. On farms, the main sources are water, cows’ udders and teat surfaces, dust, soil and milkstone deposits on farm bulk tanks and pumps, pipelines, and gaskets and processing equipment in factories [47,48]. Stewart [47] stated that the cleaning and sterilizing systems used in equipment are not very effective at eliminating spores and may even activate spores of *B. cereus*. In the factory, fouling deposits and stainless steel surfaces to which the spores can readily attach are the main sources [49,50]. Franklin [47] reported a case of a very heat-resistant *B. cereus* spore that contaminated UHT cream (processed at 140 °C for 2 s) being traced to an upstream homogenizer. At the factory, *B. cereus* may enter ESL milk from processing lines containing dead ends, pockets, corners, crevices, cracks, and joints due to the ability of their spores to readily attach to stainless steel, glass, and rubber [1,51,52]. When attached to stainless steel, spores show enhanced resistance to cleaning solutions [53]. Mugadza and Buys [28,54] reported that *Paenibacillus*, *B. pumilus*, and *B. cereus* were isolated from both filler nozzles and ESL milk; in the case of *B.* *cereus* there was a close relationship between isolates from the ESL milk and those from the filler nozzles. This indicates the importance of biofilms on equipment in the contamination of milk by *B. cereus*. Biofilms are surface-associated multicellular microbial communities embedded in an extracellular polysaccharide matrix; they are difficult to remove by normal cleaning procedures [4] (pp. 238–240).

Several authors have reported a seasonal effect on the incidence of *B. cereus* spores in milk. However, the reports are inconsistent. Where cows are housed during the winter, high spore counts are often encountered and attributed to contamination from bedding and fodder. However, Stewart [47] and Bartoszewiez et al. [55] observed higher counts during spring and summer when cows are not housed. Stewart [47] proposed that this may be due to the greater amount of dust during the summer. Slaghuis et al. [56] reported that the milk from cows grazing on pasture during summer contained more *B. cereus* than milk from cows that were housed and fed conserved feed; this suggests that pasture may be a reservoir for *B. cereus* spores.

● Pathogenicity:

The major interest in *B. cereus* from a public health perspective is related to the production of enterotoxins by several strains and its potential to cause two types of illness, diarrheal and emetic syndromes. The two types of toxins are very different. The diarrheagenic toxins are proteins with molecular weights in the range 38,000–46,000 Da. They are produced by actively growing cells and are thermolabile, being inactivated by heating at 56 °C for 30 min. Two of three forms of the diarrheagenic toxins are believed to cause food poisoning in humans. In contrast, the emetic toxin is a cyclic peptide, cereulide, with a molecular weight of 1200, which is extremely resistant to heat, surviving heating at 126 °C for 90 min. Psychrotrophic strains of *B. cereus* growing at low temperature do not produce the emetic toxin but may produce the diarrhoeagenic toxins, albeit slowly and in low concentrations [29].

Despite its widespread presence in milk and milk-based products, *B. cereus* has been implicated in very few cases of illness; however, one outbreak in the Netherlands in which pasteurized milk was implicated involved 280 patients [39]. Several authors have sounded a warning of the potential for this organism to cause disease [53,57] although it has been suggested that toxin-producing strains of *B. cereus* in milk and milk products are unlikely to cause food poisoning as their production of toxin, even at high counts, is very low [58,59]. Another reason why *B. cereus* rarely causes food poisoning is because it produces an intensely bitter flavor, making the contaminated products organoleptically unacceptable before they become toxic [11,60].

Te Giffel et al. [53] found that 28 of 37 isolates from pasteurized milk produced enterotoxin. Strains that fermented lactose produced more enterotoxin than strains that did not. Van Netten et al. [39] found that 25% of psychrotrophic *B. cereus* isolates from pasteurized milk were enterotoxin-positive. Notermans et al. [56] stated that ≥10^5^ CFU/mL of toxigenic *B. cereus* in pasteurized milk is generally considered to be a health hazard. They estimated that such numbers could be present in 7% of milk in the Netherlands at the time of consumption. However, epidemiological evidence does not indicate that *B. cereus* in milk causes disease to anywhere near this extent and hence the dose–response relationship needs to be revisited. Since the growth rate and enterotoxin production of *B. cereus* is low at 4 °C, several authors have concluded that milk or milk-based products stored at or below this temperature present a very low risk of becoming toxic [29,61], provided products are not stored for unduly long periods of time, e.g., >20 days [62]. Since ESL milk is designed to have a shelf-life of at least 30 days, it is possible for *B. cereus* to reach high counts by the end of its shelf-life if it is not destroyed by the heat process or the ESL milk is not packaged aseptically and is contaminated with the organism after the heat treatment. 

● Growth temperatures:

*B. cereus* can grow at a range of temperatures but the optimum growth temperature is generally 30–37 °C. The maximum temperature for most strains is 45–50 °C. However, some strains are capable of growing at low temperatures and these are of most concern for ESL milks. They are termed psychrotrophic (able to grow at 7 °C) or psychrotolerant (able to grow at 4 °C but not at 43 °C). The species name, *B. weihenstephanensis*, has been used for this sub-group of *B. cereus* [36]. 

In surveys, the percentage of strains capable of growing at 7 °C has varied. For example, Te Giffel et al. [63] isolated 766 *B. cereus* strains from farm environments and raw milk and found the percentage of isolates capable of growing at 7 °C was 40% and 30%, respectively. Similarly, in a survey of pasteurized milk samples, Te Giffel et al. [53] found that 53% of 106 isolates tested were psychrotrophic. However, in a survey of milk from a fluid milk processing plant and a milk powder plant, these authors found only 6% of isolates from the first and no isolates from the second plant were psychrotrophic. An interesting phenomenon observed by Mayr et al. [17] was that *B. cereus* (and three other spore-formers) grew at 8 °C after culturing at 30 °C but had not previously grown in milk at 10 °C.

For strains capable of growing at <7 °C, their growth rates decrease considerably as the temperature is decreased [44,64]. Rowan and Anderson [60] reported that of 38 psychrotrophic *B. cereus* isolates from milk-based infant formulae, one, four, and 16 isolates showed growth after 15 days at 4, 6, and 8 °C, respectively. Dufeu and Leesement [65] reported the average generation times for the four strains to be 1.3 h at 30 °C, 9.1 h at 8 °C, and 54 h at 3 °C, respectively. This compares favorably with 9.4 to 75 h (average 8.2 h) at 7 °C reported by Dufrenne et al. [66]. According to the International Dairy Federation, increasing the storage temperature of milk from 4 to 10 °C significantly enhances the growth rate. In general, storage of milk and milk products at ≤4 °C markedly reduces the risk of growth of *B. cereus* [33] and, as indicated above, would also prevent enterotoxin production [29,67]. 

● Spoilage potential

As well as being potentially pathogenic, *B. cereus* can cause substantial spoilage. It produces protease, which causes sweet curdling [68] and bitterness in pasteurized milk. The defects were noticed after 8–10 days of storage at 5–7 °C. *B. cereus* also produces phospholipase C (sometimes referred to as lecithinase), which degrades phospholipids of the milk fat globule membrane and causes fat globule coalescence, or chemical churning, resulting in defects such as bitty cream [69,70]. Lewis [59] commented that because the growth of *B. cereus* is accompanied by the production of a disagreeable odor and flavor, consumers are likely to detect spoilage well before the milk becomes a safety issue.

● Heat resistance

The heat resistance of *B. cereus* is particularly relevant to ESL milk; however, much of the information in this section is also applicable to psychrotrophic spore-formers in general. While the spores of many psychrotrophic strains are not very heat-resistant, there is actually a wide range of heat resistance amongst the spores of *B. cereus* isolates, with some strains being highly heat-resistant. Mikolajcik [71] reported that *B. cereus* (and *B. licheniformis*) produced the most heat-resistant spores in milk. Franklin [72] and Vyletelova et al. [73] showed that some *B. cereus* spores survive UHT treatment. 

Heat resistance data, *D*- and *z*-values, have been reported for the spores of several *B. cereus* spores. Unfortunately, different researchers have determined the D-values at different temperatures, which makes comparison difficult. The range of reported heat resistance data is illustrated by the following *D*-values in 21 studies that were collated by Bergere and Cerf [74]: *D*_90_, 3.6–10.8 min; *D*_95_, 0.5–20.2 min; *D*_100_, 0.3–27 min; *D*_105_, 11.2 min; *D*_110_, 11.5 min; *D*_121_, 0.03–0.04 min; (*D*-values for two very heat-tolerant strains discussed below [72,75] have been omitted from these data). Stoeckel et al. [76] later collated *D*-value data from five subsequent reports, including their own on infant formula; the range of *D*-values were as follows: *D*_90_, 1.1–12.8 min; *D*_95_, 2.0–4.4 min; *D*_100_, 0.27–1.83 min; and *D*_110_, 0.05–0.6 min. These values do not include the *D* values they obtained for spores in concentrated (50% total solids) infant formula, which were about double those of spores in standard (10% total solids) reconstituted infant formula. Van Asselt and Zweitering [77] collated 465 data points from 12 publications and determined the mean *D*_120_-value of *B. cereus* spores to be 0.041 min (2.46 s) and the upper 95% prediction interval *D*_120_-value to be 0.52 min (31.2 s). 

In about half of the studies reviewed by Bergere and Cerf [74], the heat inactivation curves were not linear; two showed shoulders and 10 showed tails. The importance of this is exemplified in a very heat-resistant *B. cereus* isolate from UHT cream that originated from an upstream homogenizer; although the majority of the spores were destroyed at 95–100 °C, a resistant fraction of ~1 in 10^5^–10^6^ survived heating at 135 °C for 4 h [72]. This small resistant fraction was sufficient to cause contamination of the UHT cream. Bradshaw et al. [75] also reported a very heat-resistant strain of *B. cereus*, but in this case no heat-resistant tail was observed. Its spores had *D*-values as follows: *D*_115.6_, 11.4 min; *D*_121.1_, 2.3 min; *D*_126.7_, 0.3 min and *D*_129.4_, 0.24 min. A report by Dufrenne et al. [66] gave the range of *D*_90_-values for spores of 11 *B. cereus* isolates as 2.2–9.2 min but one other strain had a *D*_90_-value of >100 min. Therefore *B. cereus* spores with high thermal tolerance do exist but appear to be relatively rare. Stoeckel et al. [76] commented that the spores of the *B. cereus* strain (IP5832) that they evaluated in infant formula were an example of a highly heat-resistant *B. cereus* spore and that heating processes capable of controlling it could be assumed to inactivate native spore populations in milk products. It had a *D*_100_-value of 1.83 min; it was obviously less heat-tolerant than the strains reported by Franklin, Bradshaw, and Dufrenne [66,72,75].

The range of reported *z*-values for *B. cereus* spores is 6.7–13.8 °C, with most in the range of 8–11 °C [66,74,76,77]. Hinricks and Atamer [78] (p. 716) cite the *z*-value for a reference strain as 9.4–9.7 °C, which is in the middle range of reported values. 

The above *D*- and *z*-value data for *B. cereus* spores demonstrate a wide range of heat resistance of individual strains. However, some authors have used reported *D*- and *z*-values to construct temperature–time semi-log curves of equal destruction of *B. cereus* spores. For example, van Asselt and Te Giffel [79] and de Jong et al. [11] constructed lines for a 6-log reduction, while Hinrichs and Atamer [78] constructed a 3-log reduction line. However, based on the information above, the appropriate lines for destruction of individual strains will vary considerably in both position, according to their *D*-values, and slope, according to their *z*-values. The wide range of heat tolerances of *B. cereus* spores makes the construction of representative thermal destruction curves very difficult. Published graphs should therefore be used as a guide to temperature–time combinations to use in processing but should not be assumed to apply to all strains of *B. cereus*. Interestingly, the *D*_121_-value estimated from the graphs of de Jong et al. [11] and van Asselt and Te Giffel [79] is ~0.03 s (that is, 1/6 of the 6-D of ~2 s read from the graphs) and from the graph of Hinrichs and Atamer [78] is ~0.14 s (that is, 1/3 of the 3-D of ~4.2 s read from the graph). These values however, differ considerably from those recorded, above, namely, 0.03–0.04 (1.8–2.4 s) (excluding data for very heat-tolerant strains). Bradshaw et al. [75] isolated a *B. cereus* strain that had a *D*_121.1_-value of 0.03 min (1.8 s) and a *z*-value of 7.9 °C, and commented that these *D*- and *z*-values were similar to those most commonly reported for *B. cereus*. This approximately agrees with van Asselt and Zweitering [77]: the mean *D*_120_-value of *B. cereus* spores of 0.041 min (2.46 s), (their upper 95% prediction interval *D*_120_-value was 0.52 min or 31.2 s) and a *D*_121_ reference value given by Hinricks and Atamer [78] (p. 716), of 0.04 min (2.4 s). 

An important factor in the heat resistance of *B. cereus* spores is their altered behavior when they form biofilms attached to equipment surfaces. Simmonds et al. [80] found an average increase of 205% in *D*_90_ values of three *B. cereus* strains when attached to stainless steel compared with those of planktonic cells. Similarly, Pfeifer and Kessler [81] reported increased heat resistance of *B. cereus* spores trapped between a silicone-rubber seal and a stainless-steel surface. 

● Activation, germination, and growth:

Spores of *B. cereus* have to germinate and the resulting vegetative cells grow before they can cause spoilage or produce toxin. Often spores need to be activated, for example by heat treatment, before they can germinate. However, *B. cereus* spores are able to germinate without preliminary heat treatment although the rate of germination and the proportion of spores that germinate are higher when the spores are subjected to a heat treatment [82]. *B. cereus* spores can be activated by heating in milk at temperatures in the range 65–95 °C for various times. The literature varies with regard to the optimum activation conditions, e.g., 65–75 °C [44], 74 °C for 10 s [69], 80 °C for 15 s [67], >80 °C [83], 85 °C for 2 min [84], 95 °C for 15 s [42], and 115 °C for 1 s [85]. The germination medium as well as the temperature is significant. Wilkinson and Davies [86] reported that milk heated at 65 to 75 °C for 15 s provided the best medium for germination, while Stadhouders et al. [84] found that milk heated at 94 °C for 10 s was a better germination and growth medium than HTST-pasteurized milk. 

A complication with germination of *B. cereus* spores is that they exist as both slow-germinating and fast-germinating, with the slow-germinating spores requiring more intense heat-activation treatment than the fast-germinating spores [84]. Heating fast-germinating spores of *B. cereus* in milk at 65 °C for 2 min or 72 °C for 10 s caused almost total germination at 20 °C in 24 h, while heating the slow-germinating spores at 85–90 °C for 2 min resulted in the same level of germination. It has been shown that a germinant or germination factor is produced in milk by heat treatment [34,86]. Therefore, pasteurization conditions are sufficient to cause germination of the fast-germinating spores of *B. cereus*, but not the slow-germinating spores [82,87]. Fast-germinating spores may be activated by ESL heat treatments although specific reports of this effect have not been located. These fast-germinating spores of *B. cereus* originate from soil, manure, and fodder, whereas slow-germinating spores seem to come from equipment surfaces [82]. Another issue relating to the spores with differing germination rates is that slow-germinating strains are more heat-resistant than fast-germinating strains [74]. 

Temperature has a major effect on the growth of vegetative cells following activation/germination. *B. cereus* spores may germinate at low temperatures but not show growth at these temperatures for months [47]. The abilities of *B. cereus* strains to germinate and to grow at low temperatures are not necessarily correlated. For example, Anderson Borge et al. [88] showed that, in a mixture of 11 mesophilic and psychrotolerant strains, the psychrotolerant strains exhibited both the highest and the lowest germination rates in milk at 7 and 10 °C. 

● *B. cereus* in perspective

There is no doubt that *B. cereus* is a spore-former of some concern to the dairy industry. The above discussion shows that it (or members of the *B. cereus* group in general) is widespread in the environment and a common contaminant of raw and heat-treated milk. It belongs to a group that contains eight closely related species and hence its precise identification in the reported studies cannot be assured. It has a wide range of growth temperatures amongst its strains; most strains are mesophilic but some are psychrotrophic, which means they can grow in ESL milk during refrigerated storage. The strains vary considerably in heat resistance; most are readily inactivated by common ESL heat processing but some strains are more heat-resistant. Some strains are pathogenic but produce very little toxin at low temperatures. The probability of encountering a strain of *B. cereus* whose spores are very heat-resistant and that is psychrotrophic and produces toxin at low temperature is small. However, the risk is much greater if the temperature of storage is elevated. The fact that it forms biofilms and can therefore persist on equipment means that it is a constant threat and cannot be ignored by ESL processors. 

### 3.2. Post-Processing Contamination

The above discussion relates to the keeping quality of ESL milk as affected by the bacteria in the raw milk that are not killed by the heat process. This situation applies if the milk is packaged aseptically and there is no PPC. However, ESL milk is commonly packaged in very clean, but not strictly aseptic, fillers. In this situation, special precautions are taken to minimize the risk of PPC. Such precautions typically include: use of an aseptic tank or an ultra-clean pasteurized milk holding tank equipped with sterile air blanketing; sterilization of empty final packages or packaging material with hydrogen peroxide, with or without UV irradiation; flushing of the filler with sterile air (HEPA-filtered); sterilizing the filler piping and heads with steam at 120–130 °C for 30 min; and spraying an alcohol mist into the filler before filling is commenced. However, such fillers are not completely sealed, are usually not located in a clean room, and the air in the filling zone is not completely sterile. In addition, non-sterile product is being packed in the filler, which can allow biofilm build-up if cleaning is not carried out effectively. This all amounts to the possibility, although small, of periodic contamination of the product. In fact, this is what has been reported by several authors and is the experience of processors. 

The reported bacterial contaminants in thermally-produced ESL milk packed under very clean, but non-aseptic, conditions are mostly Gram-positive. This contrasts with the situation in pasteurized milk, where Gram-negative psyschrotrophs, principally Pseudomonads, are generally the most prevalent [89]. The common spoilage organisms in commercial ESL milks included the non-spore-formers *Rhodococcus*, *Anquinibacter*, *Arthrobacter*, *Microbacterium*, *Enterococcus*, *Staphylococcus*, *Micrococcus*, and coryneforms. These bacteria appeared to enter the ESL milk from the air, the equipment, and/or the packaging material [17,89]. Mugadza and Buys [90] also isolated Gram-positive bacteria from ESL milk, namely *Arthrobacter*, *Annerococcus*, and *Mycobacterium*. 

Mugadza and Buys [24,90] also found the spore-formers *B. pumilus* (the dominant spore-former), *B. subtilus*, *Paenibacillus*, and *B. cereus* in ESL milk. *Paenibacillus* and *B. cereus*, as well as the non-spore-former *Micrococcus luteus*, were isolated from filler nozzles, which the authors proposed to be a source of contamination of the milk. As discussed above, the common occurrence of *B. cereus* in ESL milks is of concern because of its potential to be pathogenic and cause spoilage. Mugadza and Buys [28] identified several *B. cereus* isolates from ESL milk and associated equipment. Based on discriminatory polymerase chain reaction (PCR) analysis, all isolates were shown to have the csp*A* gene, indicating that they were psychrotrophs. In addition, these isolates produced proteases and hence have spoilage potential. There was a close relationship between *B. cereus* isolates from filler nozzles and those in milk, indicating the equipment as a source of contamination of the ESL milk. *Paenibacillus* has been isolated from UHT milk during a period of “tenacious periodical contamination” [26], indicating contamination from processing or packaging equipment. 

The above reports strongly suggest the involvement of biofilms in PPC. This is further supported by the fact that the contaminating bacteria in ESL milk have been identified to be processor-specific [90], which is consistent with the above discussion, where *B. circulans*, *B. licheniformis*, or *B. pumilus* was found to be the dominant organism in each of three different reports. This is similar to findings for contaminants in pasteurized milk [91,92] and suggests the need for processor-specific remedial action to reduce bacterial contamination in market milk. It is worth noting here that it has been calculated that one psychrotrophic spore per mL can cause spoilage in 18 d at 4 °C [20]. Hence a very low level of PPC in ESL milk packaged under clean but not aseptic conditions can limit the shelf-life of ESL milk. Unfortunately, such contamination can be sporadic with isolated batches and even some but not all packages in a batch being contaminated; such contamination should be eliminated by aseptic packaging. 

### 3.3. Aseptic Packaging of ESL Milk

It is apparent that the shelf-life of thermally-produced UHT milk packaged under non-aseptic conditions is limited by PPC and that it is difficult to prevent PPC in every package in every batch of ESL milk packaged in this way. Therefore, aseptic packaging deserves consideration. While aseptic packaging is usually associated with UHT products, it is recognized that it is highly desirable, though not essential, for ESL products [93,94]. Aseptic packaging operating correctly eliminates PPC and hence the shelf-life of the product is determined solely by the microorganisms that survive the heat treatment and can grow at low temperature, that is, psychrotrophic spore-formers such as *B. circulans* [19], *B. cereus*, *B. pumilus*, and *Paenbacillus* [24]. While the shelf-life of ESL milk packaged aseptically is seldom reported, it has been suggested that a refrigerated shelf-life of 90 days is possible [93]; by comparison, with very clean rather than aseptic filling, a shelf-life of 30–40 days can be expected. Brody [93] cites defect rates for ESL product packaged in ultra-clean and aseptic fillers as 1 in 1000 and 1 in 10,000, respectively. The latter is also the target for UHT milk [4] (p. 329).

Blake et al. [18] used direct UHT heating at 120–140 °C for 4 s with aseptic packaging and obtained a shelf-life of >60 days as judged by a formal taste panel. Continued sensory testing of the milk samples by a small informal panel found the milks still acceptable after 240 days. It is therefore apparent that very long refrigerated shelf-lives can be achieved with aseptic packaging and, provided the heat treatment is sufficient to inactivate the spores of any psychrotrophic pathogenic spore-formers, such as *B. cereus*, the safety of the ESL milk can be assured.

### 3.4. Storage Temperature 

The temperature of storage of the ESL milk has a major effect on its shelf-life. This was demonstrated by Rysstad and Kolstad [1], who stored pasteurized milk, which had been aseptically packaged, at 6, 8, and 10 °C. The times taken to reach a total count of 10^6^ CFU/mL were 40, 15, and 7 days, respectively. Accordingly, they recommended that ESL milk should be stored at ≤6 °C. In view of the discussion above about toxin production by *B. cereus*, storage at ≤4 °C would be preferable.

A major practical issue for ESL milk is that the temperature of the market cold chain and of consumers’ refrigerators cannot be guaranteed to be maintained at this temperature; for this reason, several researchers have carried out storage trials of ESL milk at 7 °C and even up to 10 °C [8]. The only bacteria that should grow in ESL milk are psychrotrophic bacteria, but if the temperature of storage exceeds ~6 °C, some mesophilic bacteria can grow and contribute to spoilage [89]. Such organisms could be the result of PPC or could be from heat-resistant spores surviving the ESL process. 

## 4. Optimizing the Flavor of ESL Milk

As well as the microbiological aspects, the chemical effect of the heat process also needs to be considered. The main chemical effect of concern is the production of flavor volatiles, which impart a heated or cooked flavor to the milk. The ideal ESL milk should have a flavor similar to that of pasteurized milk, that is, a very low level of heated or cooked flavor. Fortunately this is possible, although in practice the heating conditions for this to occur are seldom optimized.

At this point it is necessary to reiterate an essential difference between the kinetics of bacterial destruction and those of chemical reactions. Heating at high temperatures for short holding times favors high levels of bacterial destruction, while heating at lower temperatures for longer holding times result in high levels of chemical change. This is illustrated in a comparison of UHT processing, which typically occurs at ~140 °C for a few seconds, and in-container sterilization, which uses temperatures of 110–120 °C for 10–20 min. Both processes have a similar bactericidal effect but much more chemical change occurs in the in-container sterilized milk, as evidenced by a brownish color and a distinct cooked flavor. Similarly for ESL processing for a particular bactericidal effect, i.e., the same *F*_0_ or B*, combinations of higher temperatures for short times produce less chemical change than combinations of lower temperatures for longer times. This was illustrated by Rysstad and Kolstad [1] with a direct and an indirect process having equal bactericidal effects (i.e., the same B* or *F*_0_) (135 °C for 0.5 s and 127 °C for 1 s, respectively); the indirect process causes much more chemical change, as measured by denaturation of β-Lg, than the direct process (details below).

The chemical effect of a heat process can be assessed in several ways using the kinetics of various chemical reactions. A commonly used chemical index is C*, which is based on the kinetics of destruction of the vitamin thiamine. A C* of 1 is equivalent to a 3% destruction of thiamine and is the recommended upper limit for UHT milk. Another measure is the percentage denaturation of the whey protein, β-Lg. This is arguably a better measure than C* for ESL milk as the denaturation is accompanied by the formation of volatile sulfur compounds, which are largely responsible for the heated or cooked flavor of heat-treated milk. 

Mayer et al. [95] investigated the levels of undenatured β-Lg in commercial milk samples, including 71 ESL, obtained from retail outlets in Austria. Only 45% of the ESL milk samples analyzed had β-Lg contents of >1800 mg/L milk, a proposed limit for ESL milk [1]. (Note: an undenatured β-Lg content of 1800 mg/L represents 45–50% denaturation.) A further 55% of the analyzed ESL milk samples had low undenatured β-Lg levels, <500 mg/L, equivalent to >85% denaturation). This spread of data reflects the fact that there are no specified conditions for ESL processing.

Rysstad and Kolstad [1] reported 13.6% denaturation of β-Lg in Pure-Lac ESL milk processed at 135 °C for 0.5 s. They compared this with an indirect heating system of equal *F*_0_-value with a nominal temperature–time condition of 127 °C for 1 s, which had 83.5% denaturation. Huijs et al. [10] reported ~20–25% denaturation of β-Lg in milk treated with Innovative Steam Injection (ISI) heating at 150–180 °C for <0.1 s. 

Cooked/sulfurous flavor begins to be noticed in heated milks when denaturation of β-Lg reaches ~60% [96]. Therefore, appropriate heating conditions should be chosen in order to minimize denaturation of β-Lg and avoid development of this flavor. Therefore, the recommended level of 1800 mg/L of undenatured β-Lg [1] (45–50% denaturation) is reasonable. A percentage denaturation of 50% is proposed here as the upper limit for ESL milk. It is recognized, however, that only the undenatured level is measured and the level in the raw milk from which the ESL milk is produced is seldom known. For that reason, a level of 1600–1800 mg/mL is suggested, assuming a level of β-Lg in raw milk of 3200–3600 mg/mL. An alternative measure is the undenatured whey protein index (WPNI), which is commonly used to indicate the severity of the pre-heating process in the manufacture of skim milk powder [97]. ESL milk fits into the medium-heat range and it is proposed that the level in ESL milk should be 3.75–4.0 mg/g of dry matter. WPNI correlates very well (negatively) with denaturation of β-Lg [98].

Another chemical measure of heat load is furosine, which is a measure of the first stage of the Maillard reaction between lactose and protein-bound lysine. Lorenzen et al. [99] reported the level of furosine in commercial thermally processed ESL milk in Germany, Austria, and Switzerland to be 11.1–22.6 mg/100 g protein, while Mayer et al. [95] analyzed 71 ESL milk in Austria and found that only 45% had furosine levels <40 mg/100 g protein, a recommended upper limit. According to these authors, “good” ESL milk had a furosine level of 11.6 mg/100 g protein, while “bad” ESL milk had 71.3 mg/100 g protein. Gallman [100] reported an average furosine level for ESL milk of 20 mg/100 g protein but suggested that it should be possible to achieve <12 mg/100 g protein (and >1800 mg/L of undenatured β-Lg). 

Lactulose, which is not present in raw milk, is formed from lactose during heating and is arguably the best chemical index of heat treatment [4] (pp. 198–200). It has been used for ESL milk, with proposed values for good-quality milk of <30 mg/L [100] and <40 mg/L [8] being suggested. For reference, Pure-Lac milks were reported to have <40 mg/L [8] and commercial directly processed UHT milks have levels of ≥90 mg/L [4] (p. 199).

## 5. Optimizing ESL Heating Conditions

Unlike the temperature–time conditions for pasteurization, which are specified in most countries to be ≥72 °C for ≥15 s, there are generally no such specified conditions for ESL processing. The reported temperatures for producing ESL milk by thermal means alone vary from 90 to 145 °C. However, most ESL milk is processed at 120–130 °C, for holding times of a few seconds. In the USA, ESL milk, called “ultra-pasteurized milk,” is defined as being processed at ≥138 °C for ≥2 s. Packaging of ESL milk is commonly under ultra-clean conditions but can be aseptic [101].

Reported commercial processing conditions for ESL milk are mostly in the range 123–127 °C for 1–5 s [17,90,99,102,103,104]. Higher temperatures can also be used. As mentioned, U.S. regulations define the process of “ultra-pasteurization” as heating milk at ≥138 °C for ≥2 s. A heat treatment of 138 °C for 2 s may seem severe for ESL milk, but it is still a sub-UHT treatment with calculated *F*_0_, B*, and C* of 1.7 min, 0.4, and 0.12, respectively, for a steam infusion or steam injection system; corresponding UHT indices are ≥3 min, ≥1, and ≤1, respectively. Even higher temperatures for shorter times, up to 145 °C for <1 s, using the Pure-Lac system [1,8] and up to 180 °C for <0.1 s using the ISI system [10], have also been advocated. These higher-temperature treatments with very short holding times are still sub-UHT treatments in terms of their sporicidal effects, i.e., B* values are <1. ESL milks produced at lower temperatures are also marketed. In South Africa, ESL milk is processed at 94–100 °C [24]. This product is similar to the ESL milks discussed by Manji [105], involving processing by direct steam heating at 89–100 °C.

Several authors have reported the effects of different ESL temperature–time combinations on the shelf-life of the product. For example, Ranjith [106] showed that heating at <117.5 °C for 1 s was insufficient to prevent bacterial growth in cream during storage at 7 °C. He concluded that temperatures ≥120 °C were required for a shelf-life of 49 days at 7 °C. Blake et al. [18] found that treatments at ≤132 °C were insufficient to prevent growth during refrigerated storage but that heating at ≥134 °C for 4 s produced ESL milk with a long shelf-life. In both of these studies, the ESL milk was packaged aseptically.

**Figure 1:**
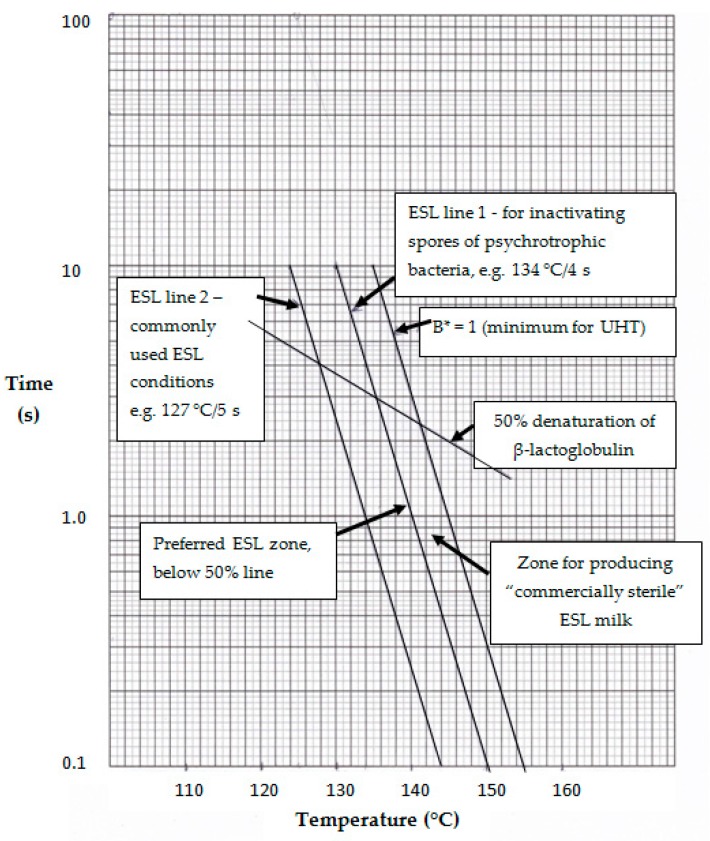
Temperature–time combinations for producing extended shelf life (ESL) and ultra-high-temperature (UHT) processed milk. Points along lines have equal effectiveness in either inactivating bacterial spores or causing 50% denaturation of β-lactoglobulin.

Based on the above discussion, it is apparent that the optimum heating conditions for producing ESL milk should be based on inactivating spores of psychrotrophic bacteria and minimizing cooked flavor. It is proposed that the former be based on the data of Blake et al. [18], which showed that treatment at ≥134 °C for 4 s was effective. This is in line with the conclusion of Bergere and Cerf [74] that milk processed at ≥134 °C should not contain *B. cereus* spores. A heat treatment 134 °C for 4 s would have a B* of 0.32 (and *F*_0_ of 1.33), considerably less than 1.0, the minimum for UHT processing [4]. This is shown in Figure 1 by “ESL line 1,” which joins points with a B* of 0.32 and is to the left of the B* = 1 line. The slope of the line is based on the assumption that the *z*-value for psychrotrophic spore inactivation is the same as for inactivation of thermophilic spores, i.e., ~10. This is a reasonable assumption given the wide range of *z*-values reported for *B. cereus* spores [66,74,76,77,78]. It is therefore proposed that the B* for ESL processing should be >0.3. 

As proposed in Section 4, the heat input for ESL milk processing should be less than that which would theoretically cause ~50% denaturation of β-Lg (according to the kinetics reported by Lyster [107]. The temperature–time conditions to achieve 50% denaturation of β-Lg are shown by the line labeled thus in Figure 1. Hence, points along the ESL line 1 below the 50% β-Lg denaturation line (the 50% line) represent the best conditions for producing ESL milk to ensure the destruction of spores of psychrotrophic bacteria and cause minimal flavor change. “ESL line 2” in Figure 1 shows temperature–time conditions equivalent to 127 for 5 s, which is representative of the conditions commonly used for ESL milk production, and joins points with a B* of ~0.09. Clearly these conditions lie to the left of ESL line 1 and would not be expected to inactivate all psychrotrophic spores. 

Points in the zone between ESL lines 1 and 2 and below the 50% line represent reasonable conditions for commercial production of ESL milk; the nearer the conditions are to ESL line 1, the more likely that spores of psychrotrophic spore-formers will be destroyed. Furthermore, points in this zone below the 50% line will have the freshest flavor. Points in the zone between ESL lines 1 and 2 and above the 50% line will have similar bactericidal effects to those below the 50% line but will have more cooked flavors. 

Milk processed at temperature–time conditions in the zones between ESL line 1 and the B* = 1 line and below the 50% line will have the greatest bacterial stability, having B* values between 0.32 and 1 and little cooked flavor. In effect, such milk could be termed “commercially sterile” ESL milk. A commercially sterile product is defined as one in which no bacterial growth occurs under the normal conditions of storage; for ESL milk, this is under refrigeration, preferably at ≤4 °C. While this term is normally applied to UHT milk, it is also applicable to ESL milk processed to inactivate all spores of psychrotrophic bacteria, and packaged aseptically. Commercial sterility implies that not all packages of every batch will be devoid of bacteria that could grow and cause spoilage. Brody [93] suggested a target defect rate of ~1 in 10,000, the same as for UHT milk [4] (p. 329).

Commercially sterile ESL milk with an expected long shelf-life has an increased risk of developing bitterness. The native milk plasmin will not be inactivated under these heating conditions and, although it has low activity at low temperature, it is not inactive [108]. De Jong [11] showed that ISI-ESL milk, a commercially sterile ESL milk that had plasmin activity, did not develop bitterness during storage at 7 °C for up to 28 days. However, bitterness may develop during longer periods of storage. In addition to plasmin, residual bacterial proteases from growth of psychrotrophic bacteria in the raw milk before processing will be more likely to cause proteolysis, and hence bitterness, during long storage times (>30–40 days) than during shorter storage times. For both plasmin and bacterial proteases, maintenance of low temperature in the cold chain, preferably at ≤4 °C, is crucial. Fluctuations to higher temperatures will increase the risk of proteolysis and the development of bitterness. Further research is required to assess the risk of bitterness development in “commercially sterile” ESL milk, with long shelf-life, from proteolysis by plasmin and bacterial proteases. 

## 6. Assessment of Some Possible Temperature–Time Conditions for ESL Processing

The predicted bactericidal (B*) and chemical (β-Lg denaturation) effects of some possible temperature–time combinations for ESL processing are shown in Table 1. These have been computed using Excel, as reported by Browning et al. [109] and Tran et al. [110], assuming the process is direct heating with a preheat temperature of 70 °C. The time assumed for reaching the required temperature from the preheat temperature of 70 °C, and also returning to 70 °C after the high-temperature holding, is 0.5 s. This illustrates the low sporicidal effects of 120 °C for 9 s and 127 °C for 5 s and the levels of β-Lg denaturation, which are higher than proposed here. By contrast, the minimum heating conditions for producing ESL (ultra-pasteurized) milk in USA, 138 °C for 2 s, has a B* of 0.4, which meets the sporicidal criterion proposed here. In Figure 1, these conditions lie just to the right of ESL line 1. This would theoretically cause ~45% denaturation of β-Lg, below the 50% line in Figure 1. Therefore, this milk meets the criteria proposed here. The conditions in the last two rows of Table 1 clearly meet the proposed criteria for both B* and β-Lg denaturation and would be expected to produce milk with a fresh flavor and long shelf-life, if packaged aseptically. These conditions are in line with recommendations from some equipment suppliers that produce systems enabling such short holding times to be achieved.

As discussed in Section 3.1.3, temperature–time lines for 6-log reduction [11,79] and 3-log reduction [78] of *B. cereus* spores have been published. If these were placed on Figure 1 they would be far to the left of ESL line 2. The first passes through the point 130 °C for 0.1 s and the second passes through the point 130 °C for 1 s. The conditions along those lines would clearly not meet the criteria for ESL milk proposed here. The approximate B* values would be 0.01 and 0.04, respectively. 

It is also instructive to indicate the position on Figure 1 for the temperature–time line of inactivation (9-log) of spores of mesophilic spore-formers. Such a line was published by Kessler [111] and reproduced by Chavan et al. [112]. It would be approximately halfway between ESL line 1 and the B* = 1 line. It would pass through the point of 135 °C for ~8 s and have a B* of ~0.8. This is clearly in excess of what is proposed here for ESL milk but, provided the temperature–time conditions fell below the 50% line in Figure 1, they would produce high-quality ESL milk. As the B* would be <1, milk processed with these conditions would not meet the recommended bactericidal criterion for UHT milk. 

In several countries, the specified temperature–time conditions for UHT processing are ≥135 °C for ≥1 s. Therefore, milk processed at the lower end of the ranges is effectively ESL milk. A direct thermal process operating at 135 °C for 1 s has a *F*_0_ of 0.45 and a B* of 0.11, which are considerably less than the minima required for a UHT process, i.e., *F*_0_ = 3 and B* = 1. However, if the ESL milk processing conditions fall within the UHT range (≥135 °C for ≥1 s.), the ESL milk may not be able to be labeled “fresh,” a practice ESL milk producers prefer to follow. 

## 7. Conclusions

To ensure the production of ESL milk with a long shelf-life and good organoleptic quality, it is proposed that the heating should be performed on a direct heating system (injection or infusion) at conditions equivalent to a B* of >0.3 that would cause ≤50% denaturation of β-Lg (i.e., at least 1600 mg/L of undenatured β-Lg remaining in the ESL milk). A further condition is that the milk should be packaged aseptically. In effect, this proposes the production of “commercially sterile” ESL milk. If a more cooked flavor is acceptable, indirect systems could be used as long as the B* for the overall process exceeds 0.3.

## Figures and Tables

**Table 1:** Theoretical bactericidal and chemical effects ^1^ of possible temperature–time combinations for producing ESL milk.

Heating Conditions (°C/s)	B*	β-Lactoglobulin Denaturation (%) ^2^	Comments
120/9	0.03	61	B* too low to inactivate spores of psychrotrophic bacteria; β-Lg denaturation too high
127/5	0.09	55	Representative of commonly used conditions for ESL milk; B* too low to inactivate spores of psychrotrophic bacteria; β-Lg denaturation marginal
134/4	0.32	56	Conditions sufficient for inactivating spores of psychrotrophic bacteria; β-Lg denaturation marginal
138/2	0.40	45	Minimum conditions for ESL in USA; meets proposed criteria
140/1	0.32	34	Meets proposed criteria; excellent conditions if short holding time can be achieved
145/0.3	0.32	24	Meets proposed criteria; excellent conditions if short holding time can be achieved

^1^ Calculated for a direct plant with preheat temperature of 70 °C and heating and cooling times between pre-heat and highest temperatures of 0.5 s each; ^2^ Based on kinetics of Lyster [107].

## References

[1] Rysstad G., Kolstad J. (2006). Conference contribution: Extended shelf life milk-advances in technology. Int. J. Dairy Technol..

[2] Schmidt V.S.J., Kaufmann V., Kulozik U., Scherer S., Wenning M. (2012). Microbial biodiversity, quality and shelf life of microfiltered and pasteurized extended shelf life (ESL) milk from Germany, Austria and Switzerland. Int. J. Food Microbiol..

[3] Khoza S. (2015). Effect of Extended Shelf Life Milk Processing on the Bacterial Composition Associated with the Nozzles of Filling Machines. Master’s Thesis.

[4] Deeth H.C., Lewis M.J. (2017). High Temperature Processing of Milk and Milk Products.

[5] Burton H. (1988). Ultra High Temperature Processing of Milk and Milk Products.

[6] Lewis M., Heppell N. (2000). Continuous Thermal Processing of Foods: Pasteurization and UHT Sterilization.

[7] Datta N., Elliott A.J., Perkins M.L., Deeth H.C. (2002). Ultra-high-temperature (UHT) treatment of milk: Comparison of direct and indirect modes of heating. Aust. J. Dairy Technol..

[8] Fredsted L.B., Rysstad G., Eie T. (1996). Pure-Lac™: The new milk with protected freshness and extended shelf life. Heat Treatments and Alternative Methods.

[9] APV (SPX) (2008). Dairy Technology.

[10] Huijs G., van Asselt A., Verdurmen R., de Jong P. (2004). High speed milk. Dairy Ind. Int..

[11] De Jong P., Britz T.J., Robinson R.K. (2008). Thermal processing of milk. Advanced Dairy Science and Technology.

[12] Van Asselt A.J., Sweere A.P.J., Rollema H.S., de Jong P. (2008). Extreme high-temperature treatment of milk with respect to plasmin inactivation. Int. Dairy J..

[13] Arofikin N.V. (2010). Liquid Product Pressure Treatment Method and Device. U.S. Patent.

[14] Myer P.R., Parker K.R., Kanach A.T., Zhu T., Morgan M.T., Applegate B.M. (2016). The effect of a novel low temperature-short time (LTST) process to extend the shelf-life of fluid milk. SpringerPlus.

[15] Noma S., Shimoda M., Hayakawa I. (2002). Inactivation of vegetative bacteria by rapid decompression treatment. J. Food Sci..

[16] Hayakawa I., Furukawa S., Midzunaga A., Horiuchi H., Nakashima T., Fujio Y., Yano Y., Ishikura T., Sasaki K. (1998). Mechanism of inactivation of heat-tolerant spores of *Bacillus stearothermophilus* IFO 12550 by rapid decompression. J. Food Sci..

[17] Mayr R., Gutser K., Busse M., Seiler H. (2004). Indigenous aerobic spore-formers in high heat treated (127 °C, 5 s) German ESL (Extended Shelf-life) milk. Milchwissenschaft.

[18] Blake M.R., Weimer B.C., McMahon D.J., Savello P.A. (1995). Sensory and microbial quality of milk processed for extended shelf life by direct steam injection. J. Food Prot..

[19] Cromie S.J., Dommett T.W., Schmidt D. (1989). Changes in the microflora of milk with different pasteurisation and storage conditions and aseptic packaging. Aust. J. Dairy Technol..

[20] Collins E.B. (1981). Heat resistant psychrotrophic microorganisms. J. Dairy Sci..

[21] Doll E.V., Scherer S., Wenning M. (2017). Spoilage of microfiltered and pasteurized extended shelf life milk is mainly induced by psychrotolerant spore-forming bacteria that often originate from recontamination. Front. Microbiol..

[22] Fromm H.I., Boor K. (2004). Characterization of pasteurized fluid milk shelf-life attributes. J. Food Sci..

[23] Ranieri M.L., Boor K.J. (2009). Short communication: Bacterial ecology of high-temperature, short-time pasteurized milk processed in the United States. J. Dairy Sci..

[24] Mugadza D.T., Buys E.M. Spoilage Potential of *Bacillus* spp. & *Paenibacillus* spp. in Extended Shelf Life (ESL) Milk. Proceedings of the SASDT 48th AGM & Symposium: Innovation and Cost Optimization.

[25] Martin N.H., Ranieri M.L., Murphy S.C., Ralyea R.D., Wiedmann M., Boor K.J. (2011). Results from raw milk microbiological tests do not predict shelf-life performance of commercially pasteurized fluid milk. J. Dairy Sci..

[26] Scheldeman P., Goossens K., Rodriguez-Diaz M., Pil A., Goris J., Herman L., De Vos P., Logan N.A., Heyndrickx M. (2004). *Paenibacillus lactis* sp. *nov*. isolated from raw and heat-treated milk. Int. J. Syst. Evol. Microbiol..

[27] Bosshard P., Zbinden R., Altwegg M. (2002). *Paenibacillus turicensis* sp. *nov*., a novel bacterium harbouring heterogeneities between 16S rRNA genes. Int. J. Syst. Evol. Microbiol..

[28] Mugadza D.T., Buys E.M. (2017). Source Tracking of *Bacillus cereus* in an Extended Shelf Life (ESL) Milk Processing Factory. http://sasdt.co.za/wp-content/uploads/2017/05/2017-Session-3-Speaker-3-Mugadza.pdf.

[29] European Food Safety Authority (EFSA) (2005). *Bacillus cereus* and other *Bacillus* spp. in foodstuffs. Opinion of the Scientific Panel on Biological Hazards on *Bacillus cereus* and other *Bacillus* spp. in foodstuffs. EFSA J..

[30] McKillip J.L. (2000). Prevalence and expression of enterotoxins in *Bacillus cereus* and other *Bacillus* spp., a literature review. Antonie Van Leeuwenhoek.

[31] Cressey P., King N., Soboleva T. Risk Profile: Bacillus cereus in Dairy Products. www.mpi.govt.nz/dmsdocument/14149.

[32] International Dairy Federation (1992). Bacillus cereus in Milk and Dairy Products.

[33] International Dairy Federation (2016). Bacillus cereus in Milk and Dairy Products.

[34] Becker H., Schaller G., von Wiese W., Terplan G. (1994). *Bacillus cereus* in infant foods and dried milk products. Int. J. Food Microbiol..

[35] Larsen H.D., Jørgensen K. (1999). Growth of *Bacillus cereus* in pasteurized milk products. Int. J. Food Microbiol..

[36] Markland S.M., Farkas D.F., Kniel K.E., Hoover D.G. (2013). Pathogenic psychrotolerant spore-formers: An emerging challenge for low-temperature storage of minimally processed foods. Foodborne Pathog. Dis..

[37] Ivy R.A., Ranieri M.L., Martin N.H., den Bakker H.C., Xavier B.M., Wiedmann M., Boor K.J. (2012). Identification and characterization of psychrotolerant spore-formers associated with fluid milk production and processing. Appl. Environ. Microbiol..

[38] Raju V.V.R., Chetty M.S., Kumar M.K. (1989). Aerobic psychrotrophic spore forming bacteria in heat treated milk. Cheiron.

[39] Van Netten P., van de Moosdijk A., van Hoensel P., Mossel D.A.A., Perales I. (1990). Psychrotrophic strains of *Bacillus cereus* producing enterotoxin. J. Appl. Bact..

[40] Champagne C.P., Laing R.R., Roy D., Mafu A.A. (1994). Psychrotrophs in dairy products: Their effect and their control. Crit. Rev. Food Sci. Nutr..

[41] Frank J.F., Doyle M.P., Beuchat L.R., Montville T.J. (1997). Milk and dairy products. Food Microbiology: Fundamentals and Frontiers.

[42] Griffiths M.W., Phillips J.D. (1990). Strategies to control the outgrowth of spores of psychrotrophic *Bacillus* spp. in dairy-products. 1. Use of naturally-occurring materials. Milchwissenschaft.

[43] Franklin J.G. (1969). Some bacteriological problems in the market milk industry in the UK. J. Soc. Dairy Technol..

[44] Coghill D., Juffs H.S. (1979). Incidence of psychrotrophic spore-forming bacteria in pasteurized milk and cream products and effect of temperature on their growth. Aust. J. Dairy Technol..

[45] Ahmed A.A.H., Moustafa M.K., Marth E.H. (1983). Incidence of *Bacillus cereus* in milk and some milk products. J. Food Prot..

[46] Rangasamy P.N., Iyer M., Roginsky H. (1993). Isolation and characterisation of *Bacillus cereus* in milk and dairy products manufactured in Victoria. Aust. J. Dairy Technol..

[47] Stewart D.B. (1975). Factors influencing the incidence of *B. cereus* spores in milk. J. Soc. Dairy Technol..

[48] Meer R.R., Baker J., Bodyfelt F.W., Griffiths M.W. (1991). Psychrotrophic *Bacillus* spp. in fluid milk-products-a review. J. Food Prot..

[49] Te Giffel M., Beumer R.R., Bonestroo M.H., Rombouts F.M. (1996). Incidence and characterization of *Bacillus cereus* in two dairy processing plants. Neth. Milk Dairy J..

[50] Salustiano V.C., Andrade N.J., Soares N.F.F., Lima J.C., Bernardes P.C., Luiz L.M.P., Fernandes P.E. (2009). Contamination of milk with *Bacillus cereus* by post-pasteurization surface exposure as evaluated by automated ribotyping. Food Control.

[51] Mugadza D.T., Buys E.M. (2017). Diversity of *Bacillus cereus* strains in extended shelf life. Int. Dairy J..

[52] Svensson B., Ekelund K., Ogura H., Christiansson A. (2004). Characterisation of *Bacillus cereus* isolated from milk silo tanks at eight different dairy plants. Int. Dairy J..

[53] Te Giffel M.C., Beumer R.R., Granum P.E., Rombouts F.M. (1997). Isolation and characterisation of *Bacillus cereus* from pasteurised milk in household refrigerators in The Netherlands. Int. J. Food Microbiol..

[54] Mugadza D.T., Buys E.M. Risk Characterisation of *Bacillus cereus* in Extended Shelf Life (ESL) Milk. Proceedings of the 21st SAAFoST International Congress and Exhibition.

[55] Bartoszewiez M., Hansen B.M., Swiecicka I. (2008). The members of the *Bacillus cereus* group are commonly present contaminants of fresh and heat-treated milk. Food Microbiol..

[56] Slaghuis B.A., Te Giffel M., Beumer R.R., Andre G. (1997). Effect of pasturing on the incidence of *Bacillus cereus* spores in raw milk. Int. Dairy J..

[57] Notermans S., Dufrenne J., Teunis P., Beumer R., Te Giffel M., Peeters Weem P. (1997). A risk assessment study of *Bacillus cereus* present in pasteurized milk. Food Microbiol..

[58] Agata N., Ohta M., Yokoyama K. (2002). Production of *Bacillus cereus* emetic toxin (cereulide) in various foods. Int. J. Food Microbiol..

[59] Jooste P.J., Anelich L.E.C.M., Britz T.J., Robinson R.K. (2008). Safety and quality of dairy products. Advanced Dairy Science and Technology.

[60] Lewis M.J. (1999). Microbiological issues associated with heat-treated milks. Int. J. Dairy Technol..

[61] Rowan N.J., Anderson J.G. (1998). Diarrhoeal enterotoxin production by psychrotrophic *Bacillus cereus* present in reconstituted milk-based infant formulae (MIF). Lett. Appl. Microbiol..

[62] Juffs H., Deeth H.C. (2007). Scientific Evaluation of Pasteurisation for Pathogen Reduction in Milk and Milk Products.

[63] Te Giffel M., Beumer R.R., Slaghuis B.A., Rombouts F.M. (1995). Occurrence and characterization of (psychrotrophic) *Bacillus cereus* on farms in The Netherlands. Neth. Milk Dairy J..

[64] Jensen I., Moir C.J., Hocking A.D. (2003). *Bacillus cereus* and other *Bacillus* species. Foodborne Microorganisms of Public Health Significance.

[65] Dufeu J., Leesment H. Growth and resistance characteristics of some psychrotrophic spore-formers isolated from raw milk. Proceedings of the XIX International Dairy Congress.

[66] Dufrenne J., Bijwaard M., Te Giffel M., Beumer R., Notermans S. (1995). Characteristics of some psychrotrophic *Bacillus cereus* isolates. Int. J. Food Microbiol..

[67] Odumeru J.A., Toner A.K., Muckle C.A., Griffiths M.W., Lynch J.A. (1997). Detection of *Bacillus cereus* diarrheal enterotoxin in raw and pasteurized milk. J. Food Prot..

[68] Overcast W.W., Atmaram K. (1974). The role of *Bacillus cereus* in sweet curdling of fluid milk. J. Milk Food Technol..

[69] Stone J.M., Rowlands A. (1952). Broken or bitty cream in raw and pasteurised milk. J. Dairy Res..

[70] Stadhouders J., Hup G. (1980). Effect of milk storage before pasteurization on the formation of flocs bitty cream by *Bacillus cereus* in pasteurized milk. Zuivelzicht.

[71] Mikolajcik E.M. (1970). Thermodestruction of *Bacillus* spores in milk. J. Milk Food Technol..

[72] Franklin J.G. (1970). Spores in milk-problems associated with UHT processing. J. Appl. Bact..

[73] Vyletelova M., Svec P., Pacova Z., Sedlacek I., Roubal P. (2002). Occurrence of *Bacillus cereus* and *Bacillus licheniformis* strains in the course of UHT milk production. Czech J. Anim. Sci..

[74] Bergere J.-L., Cerf O. (1992). Heat resistance of *Bacillus cereus* spores. Bacillus cereus in Milk and Milk Products.

[75] Bradshaw J.G., Peeler J.T., Twedt R.M. (1975). Heat-resistance of ileal loop reactive *Bacillus cereus* strains isolated from commercially canned food. Appl. Microbiol..

[76] Stoeckel M., Westermann A.C., Atamer Z., Hinrichs J. (2013). Thermal inactivation of *Bacillus cereus* spores in infant formula under shear conditions. Dairy Sci. Technol..

[77] Van Asselt E.D., Zwietering M.H. (2006). A systematic approach to determine global thermal inactivation parameters for various food pathogens. Int. J. Food Microbiol..

[78] Hinrichs J., Atamer Z., Fuquay J.W., Fox P.F. (2011). Sterilization of milk and other products. Encyclopedia of Dairy Sciences.

[79] Van Asselt A.J., Te Giffel M.C., Tamime A.Y. (2009). Hygienic practices in liquid milk dairies. Market Milks–Processing and Quality Management.

[80] Simmonds P., Mossel B.L., Intaraphan T., Deeth H.C. (2003). The heat resistance of *Bacillus* spores when adhered to stainless steel and its relationship to spore hydrophobicity. J. Food Prot..

[81] Pfeifer J., Kessler H. (1995). Heat resistance of *Bacillus* cereus spores located between seals and seal surfaces. J. Food Prot..

[82] Bergere J.-L. (1992). Spore formation and germination of *Bacillus cereus*: The spore cycle. Bacillus cereus in Milk and Milk Products.

[83] Stadhouders J., Hup G., Langeveld L.P.M. (1980). Some observations on the germination, heat resistance and outgrowth of fast-germinating and slow-germinating spores of *Bacillus cereus* in pasteurized milk. Neth. Milk Dairy J..

[84] Guirguis A.H., Griffiths M.W., Muir D.D. (1983). Spore-forming bacteria in milk. 1. Optimization of heat-treatment for activation of spores of *Bacillus* species. Milchwissenschaft.

[85] Wilkinson G., Davies F.L. (1973). Germination of spores of *Bacillus cereus* in milk and milk dialysates: Effect of heat treatment. J. Appl. Bact..

[86] Labots H., Hup G. (1964). *Bacillus cereus* in raw and pasteurized milk. II. The occurrence of slow and fast germinating spores in milk and their significance in the enumeration of *B. cereus* spores. Neth. Milk Dairy J..

[87] Anderson Borge G.I., Skeie M., Sorhaug T., Langsrud T., Granum P.E. (2001). Growth and toxin profiles of *Bacillus cereus* isolated from different food sources. Int. J. Food Microbiol..

[88] Griffiths M.W., Phillips J.D. (1988). Modeling the relation between bacterial-growth and storage-temperature in pasteurized milks of varying hygienic quality. J. Soc. Dairy Technol..

[89] Mayr R., Gutser K., Busse M., Seiler H. (2004). Gram-positive non-spore-forming bacteria are frequent spoilage organisms of German retail ESL Extended Shelf-life milk. Milchwissenschaft.

[90] Mugadza D., Buys E. (2014). Diversity of Spore Former and Non-Spore Former Bacteria in Extended Shelf-Life (ESL) Milk.

[91] Griffiths M.W., Phillips J.D. (1986). The application of the preincubation test in commercial dairies. Aust. J. Dairy Technol..

[92] Craven H.M., Macauley B.J. (1992). Micro-organisms in pasteurised milk after refrigerated storage. 3. Effect of milk processors. Aust. J. Dairy Technol..

[93] Brody A.L. (2006). Aseptic and extended-shelf-life packaging. Food Technol..

[94] Brody A.L. (2000). The when and why of aseptic packaging. Food Technol..

[95] Mayer H.K., Raba B., Meier J., Schmid A. (2010). RP-HPLC analysis of furosine and acid-soluble β-lactoglobulin to assess the heat load of extended shelf life milk samples in Austria. Dairy Sci. Technol..

[96] Gaafar A.M.M. (1987). Investigation into the Cooked Flavor in Heat-Treated Milk. Ph.D. Thesis.

[97] Oliveira L.N., Marinho V.T., Zamagno M.V., Lauro M.A., Barboza J.A.N., da Silva P.H.F. (2015). Assessment of whey protein nitrogen index as an indicator of heat treatment for UHT milk and milk powder. J. Candido Tostes Dairy Inst..

[98] Anema S.G., Lloyd R.J. (1999). Analysis of whey protein denaturation: A comparative study of alternative methods. Milchwissenschaft.

[99] Lorenzen P.C., Clawin-Raedecker I., Einhoff K., Hammer P., Hartmann R., Hoffmann W., Martin D., Molkentin J., Walte H.G., Devrese M. (2011). A survey of the quality of extended shelf life (ESL) milk in relation to HTST and UHT milk. Int. J. Dairy Technol..

[100] Gallmann P.U. (2000). Possible quality improvements/benefits of extended shelf life (ESL) milk. Proceedings of the Conference on Extended Shelf-Life Milk.

[101] Kaufmann V., Scherer S., Kulozik U. (2010). Procedures for the prolongation of the storability of consumable milk and its material changes: ESL milk. J. Verbraucherschutz Lebensm.-J. Consum. Prot. Food Saf..

[102] Vatne K.B., Castberg H.B. (1991). Processing and packaging aspects of extended shelf life products. Aust. J. Dairy Technol..

[103] Kaufmann V., Kulozik U. (2008). Processing factors influencing quality and stability of extended shelf life (ESL) milk. DMZ Lebensm. Milchmirtschaft.

[104] MGT ESL Systems. Extended Shelf Life Milk (Brochure). http://www.mgt.co.il/sites/MGT_site/UserContent/files/MGT_ESL_brochure_V9.pdf.

[105] Manji B. (2000). Regulatory perspectives to ESL products: North American situation. Proceedings of the Conference on Extended Shelf-Life Milk.

[106] Ranjith H.M.P. (2000). High temperature pasteurization. Proceedings of the Conference on Extended Shelf-Life Milk.

[107] Lyster R.L.J. (1970). The denaturation of α-lactalbumin and β-lactoglobulin in heated milk. J. Dairy Res..

[108] Crudden A., Fox F., Kelly A.L. (2005). Factors affecting the hydrolytic action of plasmin in milk. Int. Dairy J..

[109] Browning E., Lewis M., MacDougall D. (2001). Predicting safety and quality parameters for UHT-processed milks. Int. J. Dairy Technol..

[110] Tran H., Datta N., Lewis M.J., Deeth H.C. (2008). Predictions of some product parameters based on the processing conditions of ultra-high-temperature milk plants. Int. Dairy J..

[111] Kessler H.G. (1981). Food Engineering and Dairy Technology.

[112] Chavan R.S., Chavan S.R., Khedkar C.D., Jana A.H. (2011). UHT milk processing and effect of plasmin activity on shelf-life: A review. Comp. Rev. Food Sci. Food Saf..

